# Energy, Exergetic, and Thermoeconomic Analyses of Hydrogen-Fueled 1-kW Proton-Exchange Membrane Fuel Cell

**DOI:** 10.3390/e26070566

**Published:** 2024-06-30

**Authors:** Yungpil Yoo, Sang-Yup Lee, Seok-Ho Seo, Si-Doek Oh, Ho-Young Kwak

**Affiliations:** 1 Department of Climate Change Energy Engineering, Yonsei University, Seoul 03722, Republic of Korea; yyp@besico.co.kr (Y.Y.); leessy@yonsei.ac.kr (S.-Y.L.); 2 Blue Economy Strategy Institute Co., Ltd., #602, 150 Dogok-ro, Gangnam-gu, Seoul 06260, Republic of Korea; shseo@besico.co.kr (S.-H.S.); ohsidoek@besico.co.kr (S.-D.O.); 3 Department of Chemical and Biomolecular Engineering, Yonsei University, Seoul 03722, Republic of Korea; 4 Department of Mechanical Engineering, Chung-Ang University, Seoul 06974, Republic of Korea

**Keywords:** entropy generation rate, Gouy–Stodolar theorem, proton-exchange membrane fuel cell (PEMFC), lost cost flow rate

## Abstract

Exergy analysis evaluates the efficiency of system components by quantifying the rate of entropy generation. In general, the exergy destruction rate or irreversibility rate was directly obtained through the exergy balance equation. However, this method cannot determine the origin of the component’s entropy generation rate, which is a very important factor in system design and improvement. In this study, a thorough energy, exergy, and thermoeconomic analysis of a proton-exchange membrane fuel cell (PEMFC) was performed, providing the heat transfer rate, entropy generation rate, and cost loss rate of each component. The irreversibility rate of each component was obtained by the Gouy–Stodola theorem. Detailed and extensive exergy and thermoeconomic analyses of the PEMFC system determined that water cooling units experience the greatest heat transfer among the components in the studied PEMFC system, resulting in the greatest irreversibility and, thus, the greatest monetary flow loss.

## 1. Introduction

Proton-exchange membrane fuel cells (PEMFCs) fueled by green hydrogen are suitable for small-scale distributed power generation because of their high electrical efficiency at low operating temperatures and zero emission of air pollutants [1]. In addition, the PEMFC cooling, heating, and power (CHP) system [2,3], which has a fast response to load changes, was confirmed to be the most effective system for residential use through dynamics analysis [4]. For more effective residential applications, a combined cooling, heating, and power (CCHP) system that integrates absorption cooling systems and low- and high-temperature PEMFC systems have been proposed [5]. For instance, a CCHP system integrated with a high-temperature PEMFC was proposed for data center applications with a constant cooling demand [6]. A demonstration of a residential CHP system based on a PEMFC was performed by Gigliucci et al. [1], and the seasonal optimal operation of a 1 kW PEMFC-based cogeneration system in an apartment complex was also determined [7]. As surveyed above, the applicability of various types of PEMFC to our daily lives is increasing, and accordingly, efficiency analysis of PEMFC components is required.

Exergy analysis predicts the thermodynamic performance of an energy system and the efficiency of system components by accurately quantifying the entropy generation of components [8]. Therefore, it is essential to utilize exergy analysis for thermal systems to improve the efficiency of components or to identify the origins of inefficiencies in components. Recently, exergy analysis has been applied in the diagnosis of defective components in power plants [9,10,11], PEMFC systems [12], and air conditioning systems [13,14]. In previous exergy analyses [10,15], the amount of exergy destruction of a component was simply calculated by subtracting the product (output) exergy from the fuel (input) exergy. However, heat transfer interactions are found to play an important role in increasing the irreversibility of malfunction cases in PEMFC systems [12]. Meanwhile, the difference between entropy inflow and outflow plays an important role in increasing the irreversibility of the devices in power plants where heat transfer from components to the environment is negligible [16].

All the components of power plants, such as combined-cycle power plants [16], coal-fired power plants [8], absorption refrigeration systems [6], and PEMFC systems [12], can be treated as being in thermal contact with an environment with a temperature T_o_. For these components, the exergy destruction or irreversibility rate can be obtained using the first and second laws of thermodynamics, which will be clearly explained in this paper. However, this approach results in a negative irreversibility rate in a component of PEMFC system [12]. Such a negative irreversibility rate also occurred in the generator and absorber of LiBr-water absorption refrigeration systems [6] and in the heat sink of ground-source heat pump systems [17]. Negative irreversibility may occur in components where the heat input into the component is greater than the irreversibility rate due to the difference between entropy flowing out and entropy flowing in. 

In this study, the heat transfer rate and entropy generation of a component were evaluated using the first and second laws of thermodynamics, respectively. The entropy generation rate, which can be used to calculate the irreversibility rate through the Gouy–Stodolar theorem [18], can be obtained from the entropy flow into and out of the component and the heat transfer between the component and the surroundings. All components of the PEMFC system are assumed to be in thermal contact with the environment at temperature T_o_. Additionally, a detailed exergetic and thermoeconomic analysis of the PEMFC system can identify the components that lose the largest monetary flow due to irreversibility rates, and these findings can suggest modifications to the system.

## 2. Energy and Exergy Analyses of Thermal Systems

### 2.1. Energy Conservation

The first law of thermodynamics for any thermal component at the steady flow condition may be written as follows:

(1) $${\dot{Q}}_{cv}+\sum_{in}{\dot{H}}_{i}=\sum_{out}{\dot{H}}_{i}+{\dot{W}}_{cv}$$

where ${\dot{Q}}_{cv}$ denotes the heat transfer interaction between a component and the environment, ${\dot{H}}_{i}=\dot{m}h_{i}$ is the enthalpy flow rate of *i* species at the inlet or outlet, and ${\dot{W}}_{cv}$ is the workflow rate in the component. The amount of heat transfer, i.e., ${\dot{Q}}_{cv}$, which is not usually measured, can be obtained from Equation (1) using known values of the enthalpy flow rates at the inlet and outlet and the workflow rate.

### 2.2. Exergy Balance Equation and the Second Law of Thermodynamics

A general exergy balance equation that can be applied to any component of thermal systems was formulated by Oh et al. [19]. By taking into account exergy losses due to heat transfer through non-adiabatic components to the environment with temperature of T_o_ and separating the material flow into thermal and mechanical exergy flows [19], the general exergy balance equation can be written as follows:

(2) $${\dot{E}}_{x}^{CHE}+(\sum_{inlet}{\dot{E}}_{x,i}^{T}-\sum_{outlet}{\dot{E}}_{x,i}^{T})+(\sum_{inlet}{\dot{E}}_{x,i}^{P}-\sum_{outlet}{\dot{E}}_{x,i}^{P})+T_{o}(\sum_{inlet}{\dot{S}}_{i}-\sum_{outlet}{\dot{S}}_{i}+{\dot{Q}}_{cv}/T_{o})={\dot{E}}_{x}^{W}$$

where ${\dot{E}}_{x,i}(=\dot{m}e_{x,i})$ and ${\dot{S}}_{i}(={\dot{m}}_{i}s_{i})$ denote the exergy or entropy flow rates of i species, respectively, and T_o_ is ambient temperature. The superscripts CHE, T, P, and W represent chemical, thermal, and mechanical exergy and work, respectively. The terms of ${\dot{E}}_{x}^{CHE}$ and ${\dot{E}}_{x}^{W}$ denote the exergy flow rate of the fuel and workflow rate, respectively. The fourth term is the negative value of the rate of work loss or irreversibility due to entropy generation. The exergy-balance equation given in Equation (2) can be obtained by directly combining the first and second laws of thermodynamics, and can also be generalized to a system in thermal contact with r heat reservoirs with temperature T_r_ (See Appendix A).

The entropy generation rate in the exergy balance equation was obtained using the second law of thermodynamics under steady-state flow conditions, as follows:

(3) $${\dot{S}}_{gen}=\sum_{outlet}{\dot{S}}_{i}-\sum_{inlet}{\dot{S}}_{i}-{\dot{Q}}_{cv}/T_{o}$$

The irreversibility rate $\dot{I}$ can be obtained from the Gouy–Stodola theorem [18], as follows:

(4) $$\dot{I}=T_{o}{\dot{S}}_{gen}=T_{o}\left(\sum_{out}{\dot{S}}_{i}-\sum_{in}{\dot{S}}_{i}\right)-{\dot{Q}}_{cv}$$

This equation states that the exergy destruction rate is directly related to the rate of entropy flow into and out of the component (entropy flow) and the rate of heat transfer from the component to the environment (heat transfer).

If we do not separate the exergy flow into thermal and mechanical parts, the exergy balance equation shown in Equation (2) is simplified to the following:

(5) $${\dot{E}}_{x}^{CHE}+(\sum_{inlet}{\dot{E}}_{x,i}-\sum_{outlet}{\dot{E}}_{x,i})+T_{o}(\sum_{inlet}{\dot{S}}_{i}-\sum_{outlet}{\dot{S}}_{i}+{\dot{Q}}_{cv}/T_{o})={\dot{E}}_{x}^{W}$$

The exergy appeared in Equations (2) and (5) can be defined as follows:

(6) $${\dot{E}}_{x}=\dot{m}e_{x}=\dot{m}\left[\left(h-T_{o}s\right)-\left(h_{o}-T_{o}s_{o}\right)\right]$$

where the subscript “*o*” denotes reference values, usually taken as ambient temperature (T_o_) and pressure (P_o_). 

By substituting the exergy definition into the exergy balance equation given in Equation (5), the exergy balance equation becomes the first law of thermodynamics. This outcome is rational because the exergy balance equation can be obtained directly from the first and second laws of thermodynamics (see the Appendix A).

The exergy balance equation given in Equation (5) can be rewritten as follows:

(7) $${\dot{E}}_{x}^{CHE}+\sum_{inlet}{\dot{E}}_{x,i}=\sum_{outlet}{\dot{E}}_{x,i}+{\dot{E}}_{x}^{W}+{\dot{E}}_{x}^{D}$$

Therefore, the irreversibility rate, i.e., $\dot{I}$, or exergy destruction rate, i.e., ${\dot{E}}_{x}^{D}$, can be determined by Equation (7) without information on the heat transfer of the components. In previous exergy studies, the irreversibility rate was obtained through the above exergy balance equation. The left side of Equation (7) represents “fuel” or input exergy, and the right side represents “product’ or output exergy.

The exergy stream defined in Equation (6) is calculated using Equation (8), including enthalpy (*h*) and entropy (*s*) per unit mass.

(8) $${\dot{E}}_{x}=\dot{m}e_{x}=\dot{m}\left\{\left[h(T,P)-h_{o}(T_{o},P_{o})\right]-T_{o}\left[s(T,P)-s_{o}(T_{o},P_{o})\right]\right\}$$

where *T* is temperature and *P* is pressure. The exergy stream per unit mass (ex) can be further recognized as the combination of its thermal and mechanical components, which are described as the superscripts T and P, respectively, as follows [20]:

(9) $$e_{x}=e_{x}^{T}+e_{x}^{P}$$

and

(10) $$e_{x}^{T}=\left[h(T,P)-h(T_{o},P)\right]-T_{o}\left[s(T,P)-s(T_{o},P)\right]$$

(11) $$e_{x}^{P}=\left[h(T_{o},P)-h_{o}(T_{o},P_{o})\right]-T_{o}\left[s(T_{o},P)-s_{o}(T_{o},P_{o})\right]$$

The energy conservation equation and exergy balance equation for each component of the hydrogen-fueled 1 kW PEMFC system can be obtained by applying Equations (1) and (2), respectively.

### 2.3. Exergy Cost-Balance Equation 

In the modified production structure analysis (MOPSA) method [16], a cost-balance equation corresponding to the exergy balance equation can be obtained by assigning a unit cost to each exergy flow. For example, the unit cost of C_o_ is assigned to the chemical exergy flow. The resulting exergy cost-balance equation corresponding to the exergy balance equation is given by Equation (2), as follows:

(12) $${\dot{E}}_{x}^{CHE}C_{o}+(\sum_{inlet}{\dot{E}}_{x,i}^{T}-\sum_{outlet}{\dot{E}}_{x,i}^{T})C_{T}+(\sum_{inlet}{\dot{E}}_{x,i}^{P}-\sum_{outlet}{\dot{E}}_{x,i}^{P})C_{P}+T_{o}(\sum_{inlet}{\dot{S}}_{i}-\sum_{outlet}{\dot{S}}_{i}+{\dot{Q}}_{cv}/T_{o})C_{S}+\dot{Z}={\dot{E}}_{x}^{W}C_{W}$$

where *C_T_* and *C_P_* are the unit costs of thermal and mechanical exergies, respectively, *C_S_* is the unit cost of lost work and *C_W_* is the unit cost of work or electricity. When Equation (12) is applied to a component of a thermal system to obtain the exergy cost-balance for that device, a new unit cost is assigned to the main product of that component and is displayed in Gothic letters. In Equation (12), ${\dot{Z}}_{i}$ is the flow of capital cost per unit time, including the initial purchase cost and operating cost of the i-th component, and can be obtained by the following equation [21]:

(13) $${\dot{Z}}_{i}=\phi_{i}\cdot {\dot{C}}_{i}/\delta_{i}$$

In Equation (13), $\phi_{i}$ and $\delta_{i}$ are the maintenance factor and the annual operating hours, respectively. ${\dot{C}}_{i}$ is the initial purchase cost of the equipment converted into an annual equivalent cost and is given as follows:

(14) $${\dot{C}}_{i}=\left[C_{i}-SV\cdot PWF(i,n)\right]\cdot CRF(i,n)$$

In Equation (14), *C_i_* is the initial purchase cost of the equipment, *SV* is the depreciation cost after n years of the life of the equipment, and *PWF* and *CRF* are the present worth factor and capital recovery factor, respectively. Maintenance costs are taken into account with a coefficient of $\phi_{i}$ = 1.06 for each component, assuming an expected life of 10 years. However, the lifespan of the fuel cell predicted based on the first-order kinetic model [22] was found to be much shorter than the assumed lifespan of the PEMFC. The annual interest rate is assumed to be 5% in this study.

## 3. Hydrogen-Fueled 1-kW PEMFC System

**Figure 1:**
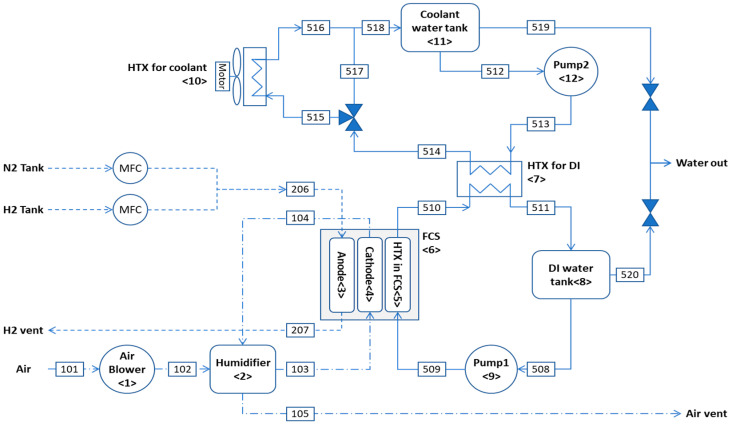
Schematic of hydrogen-fueled 1-kW PEMFC system.

A schematic of the hydrogen-fueled 1-kW PEMFC system considered is shown in Figure 1. The main system consists of nine components: an air blower <1>, a humidifier <2>, an anode <3>, a cathode <4>, a heat exchanger (HTX) for the fuel cell stack (FCS) <5>, an FCS <6>, an HTX for deionized (DI) water <7>, a DI water tank <8>, and a pump <9>. To cool the DI water heated in the FCS, a cooling device consisting of the HTX for the coolant <10>, a coolant tank <11>, and a pump <12> is used. The FCS is an artificial device that generates power and heat through an electrochemical reaction with hydrogen.

### 3.1. Energy Conservation and Exergy Balance and Exergy Cost-Balance Equations for PEMFC System

The following energy conservation and exergy balance equations can be obtained by applying the first law of thermodynamics shown in Equation (1) and the general exergy balance equation given in Equation (2) to each component in the hydrogen-fueled PEMFC system. The exergy cost-balance equation can be obtained by applying Equation (12) to each component. In the descriptions of both exergy and cost-balance, the first digit in the subscript indicates a specific fluid stream: 1 for air, 2 for hydrogen, and 5 for water. The second and third digits represent the inlet and outlet of points in the components. The number in the bracket < > indicates each component of the PEMFC system. In the exergy cost-balance equation, the number in the subscript of the unit cost refers to the component, and the unit cost of a specific exergy expressed in Gothic font represents the unit cost of the main product of the component. For each component of the PEMFC, the first law of thermodynamics and the exergy balance and exergy cost-balance equations are as follows:

1. Air blower
  (15-1) $${\dot{H}}_{101}+{\dot{Q}}_{<1>}={\dot{H}}_{102}+{\dot{W}}_{<1>}$$
  (15-2) $$\begin{array}{l} \left({\dot{E}}_{x,101}^{T}-{\dot{E}}_{x,102}^{T}\right)+\left({\dot{E}}_{x,101}^{P}-{\dot{E}}_{x,102}^{P}\right)+\left({\dot{E}}_{x,101}^{C}-{\dot{E}}_{x,102}^{C}\right)\\ +\left[T_{o}\left({\dot{S}}_{101}-{\dot{S}}_{102}\right)+{\dot{Q}}_{<1>}\right]={\dot{E}}_{x,<1>}^{W} \end{array}$$
  (15-3) $$\begin{array}{c} \left({\dot{E}}_{x,101}^{T}-{\dot{E}}_{x,102}^{T}\right)C_{T}+\left({\dot{E}}_{x,101}^{P}-{\dot{E}}_{x,102}^{P}\right)C_{1P}+\left({\dot{E}}_{x,101}^{C}-{\dot{E}}_{x,102}^{C}\right)C_{C}\\ +\left[T_{o}\left({\dot{S}}_{101}-{\dot{S}}_{102}\right)+{\dot{Q}}_{<1>}\right]C_{S}+{\dot{Z}}_{<1>}={\dot{E}}_{x,<1>}^{W}C_{W} \end{array}$$
  where ${\dot{W}}_{<1>}={\dot{E}}_{x,<1>}^{W}$.
2. Humidifier
  (16-1) $${\dot{H}}_{102}+{\dot{H}}_{104}+{\dot{Q}}_{<2>}={\dot{H}}_{103}+{\dot{H}}_{105}$$
  (16-2) $$\begin{array}{l} \left[\left({\dot{E}}_{x,102}^{T}-{\dot{E}}_{x,103}^{T}\right)+\left({\dot{E}}_{x,104}^{T}-{\dot{E}}_{x,105}^{T}\right)\right]+\left[\left({\dot{E}}_{x,102}^{P}-{\dot{E}}_{x,103}^{P}\right)+\left({\dot{E}}_{x,104}^{P}-{\dot{E}}_{x,105}^{P}\right)\right]\\ +\left[\left({\dot{E}}_{x,102}^{C}-{\dot{E}}_{x,103}^{C}\right)+\left({\dot{E}}_{x,104}^{C}-{\dot{E}}_{x,105}^{C}\right)\right]+\left[T_{o}\left({\dot{S}}_{102}-{\dot{S}}_{103}+{\dot{S}}_{104}-{\dot{S}}_{105}\right)+{\dot{Q}}_{<2>}\right]=0 \end{array}$$
  (16-3) $$\begin{array}{l} \left[\left({\dot{E}}_{x,102}^{T}-{\dot{E}}_{x,103}^{T}\right)+\left({\dot{E}}_{x,104}^{T}-{\dot{E}}_{x,105}^{T}\right)\right]C_{2T}+\left[\left({\dot{E}}_{x,102}^{P}-{\dot{E}}_{x,103}^{P}\right)+\left({\dot{E}}_{x,104}^{P}-{\dot{E}}_{x,105}^{P}\right)\right]C_{P}\\ +\left[\left({\dot{E}}_{x,102}^{C}-{\dot{E}}_{x,103}^{C}\right)+\left({\dot{E}}_{x,104}^{C}-{\dot{E}}_{x,105}^{C}\right)\right]C_{C}+\left[T_{o}\left({\dot{S}}_{102}-{\dot{S}}_{103}+{\dot{S}}_{104}-{\dot{S}}_{105}\right)+{\dot{Q}}_{<2>}\right]C_{S}\\ +{\dot{Z}}_{<2>}=0 \end{array}$$
3. Anode
  (17-1) $${\dot{H}}_{206}+{\dot{Q}}_{<3>}={\dot{H}}_{207}$$
  (17-2) $$\left({\dot{E}}_{x,206}^{T}-{\dot{E}}_{x,207}^{T}\right)+\left({\dot{E}}_{x,206}^{P}-{\dot{E}}_{x,207}^{P}\right)+\left[T_{o}({\dot{S}}_{206}-{\dot{S}}_{207})+{\dot{Q}}_{<3>}\right]=0$$
  (17-3) $$\left({\dot{E}}_{x,206}^{T}-{\dot{E}}_{x,207}^{T}\right)C_{3T}+\left({\dot{E}}_{x,206}^{P}-{\dot{E}}_{x,207}^{P}\right)C_{P}+\left[T_{o}({\dot{S}}_{206}-{\dot{S}}_{207})+{\dot{Q}}_{<3>}\right]C_{S}+{\dot{Z}}_{<3>}=0$$
4. Cathode
  (18-1) $${\dot{H}}_{103}+{\dot{Q}}_{<4>}={\dot{H}}_{104}$$
  (18-2) $$\left({\dot{E}}_{x,103}^{T}-{\dot{E}}_{x,104}^{T}\right)+\left({\dot{E}}_{x,103}^{P}-{\dot{E}}_{x,104}^{P}\right)+\left({\dot{E}}_{x,103}^{C}-{\dot{E}}_{x,104}^{C}\right)+\left[T_{o}({\dot{S}}_{103}-{\dot{S}}_{104})+{\dot{Q}}_{<4>}\right]=0$$
  (18-3) $$\begin{array}{l} \left({\dot{E}}_{x,103}^{T}-{\dot{E}}_{x,104}^{T}\right)C_{T}+\left({\dot{E}}_{x,103}^{P}-{\dot{E}}_{x,104}^{P}\right)C_{P}+\left({\dot{E}}_{x,103}^{C}-{\dot{E}}_{x,104}^{C}\right)C_{4C}\\ +\left[T_{o}({\dot{S}}_{103}-{\dot{S}}_{104})+{\dot{Q}}_{<4>}\right]C_{S}+{\dot{Z}}_{<4>}=0 \end{array}$$
5. HTX in the FCS
  (19-1) $${\dot{H}}_{509}+{\dot{Q}}_{<5>}={\dot{H}}_{510}$$
  (19-2) $$\left({\dot{E}}_{x,509}^{T}-{\dot{E}}_{x,510}^{T}\right)+\left({\dot{E}}_{x,509}^{P}-{\dot{E}}_{x,510}^{P}\right)+\left[T_{o}({\dot{S}}_{509}-{\dot{S}}_{510})+{\dot{Q}}_{<5>}\right]=0$$
  (19-3) $$\left({\dot{E}}_{x,509}^{T}-{\dot{E}}_{x,510}^{T}\right)C_{5T}+\left({\dot{E}}_{x,509}^{P}-{\dot{E}}_{x,510}^{P}\right)C_{DP}+\left[T_{o}({\dot{S}}_{509}-{\dot{S}}_{510})+{\dot{Q}}_{<5>}\right]C_{S}+{\dot{Z}}_{<5>}=0$$
6. FCS
  (20-1) $$P_{therm}={\dot{E}}_{x}^{CHE}\left(={\dot{E}}_{x,in}^{CHE}-{\dot{E}}_{x,out}^{CHE}\right)-{\dot{E}}_{x,<6>}^{W}$$
  (20-2) $${\dot{E}}_{x,<6>}^{D}=\eta P_{therm}$$
  (20-3) $${\dot{E}}_{x}^{CHE}C_{o}+(P_{them}-{\dot{E}}_{x}^{D})C_{T}+{\dot{E}}_{x}^{D}C_{S}+{\dot{Z}}_{<6>}={\dot{E}}_{x,<6>}^{W}C_{6W}$$
7. HTX for DI
  (21-1) $${\dot{H}}_{510}+{\dot{H}}_{513}+{\dot{Q}}_{<7>}={\dot{H}}_{511}+{\dot{H}}_{514}$$
  (21-2) $$\begin{array}{c} \left({\dot{E}}_{x,510}^{T}-{\dot{E}}_{x,511}^{T}\right)+\left({\dot{E}}_{x,513}^{T}-{\dot{E}}_{x,514}^{T}\right)+\left({\dot{E}}_{x,510}^{P}-{\dot{E}}_{x,511}^{P}\right)+\left({\dot{E}}_{x,513}^{P}-{\dot{E}}_{x,514}^{P}\right)+\\ \left[T_{o}\left({\dot{S}}_{510}-{\dot{S}}_{511}+{\dot{S}}_{513}-{\dot{S}}_{514}\right)+{\dot{Q}}_{<7>}\right]=0 \end{array}$$
  (21-3) $$\begin{array}{c} \left[\left({\dot{E}}_{x,510}^{T}-{\dot{E}}_{x,511}^{T}\right)+\left({\dot{E}}_{x,513}^{T}-{\dot{E}}_{x,514}^{T}\right)\right]C_{7T}+\left[\left({\dot{E}}_{x,510}^{P}-{\dot{E}}_{x,511}^{P}\right)+\left({\dot{E}}_{x,513}^{P}-{\dot{E}}_{x,514}^{P}\right)\right]C_{P}+\\ \left[T_{o}\left({\dot{S}}_{510}-{\dot{S}}_{511}+{\dot{S}}_{513}-{\dot{S}}_{514}\right)+{\dot{Q}}_{<7>}\right]C_{S}+{\dot{Z}}_{<7>}=0 \end{array}$$
8. DI water tank
  (22-1) $${\dot{H}}_{511}+{\dot{Q}}_{<8>}={\dot{H}}_{508}$$
  (22-2) $$\left({\dot{E}}_{x,511}^{T}-{\dot{E}}_{x,508}^{T}\right)+\left({\dot{E}}_{x,511}^{P}-{\dot{E}}_{x,508}^{P}\right)+\left[T_{o}\left({\dot{S}}_{511}-{\dot{S}}_{508}\right)+{\dot{Q}}_{<8>}\right]=0$$
  (22-3) $$\left({\dot{E}}_{x,511}^{T}-{\dot{E}}_{x,508}^{T}\right)C_{8T}+\left({\dot{E}}_{x,511}^{P}-{\dot{E}}_{x,508}^{P}\right)C_{P}+\left[T_{o}\left({\dot{S}}_{511}-{\dot{S}}_{508}\right)+{\dot{Q}}_{<8>}\right]C_{S}+{\dot{Z}}_{<8>}=0$$
9. Pump 1
  (23-1) $${\dot{H}}_{508}+{\dot{Q}}_{<9>}={\dot{H}}_{509}+{\dot{W}}_{<9>}$$
  (23-2) $$\left({\dot{E}}_{x,508}^{T}-{\dot{E}}_{x,509}^{T}\right)+\left({\dot{E}}_{x,508}^{P}-{\dot{E}}_{x,509}^{P}\right)+\left[T_{o}\left({\dot{S}}_{508}-{\dot{S}}_{509}\right)+{\dot{Q}}_{<9>}\right]={\dot{E}}_{x,<9>}^{W}$$
  (23-3) $$\left({\dot{E}}_{x,508}^{T}-{\dot{E}}_{x,509}^{T}\right)C_{T}+\left({\dot{E}}_{x,508}^{P}-{\dot{E}}_{x,509}^{P}\right)C_{9P}+\left[T_{o}\left({\dot{S}}_{508}-{\dot{S}}_{509}\right)+{\dot{Q}}_{<9>}\right]C_{S}+{\dot{Z}}_{<9>}={\dot{E}}_{x,<9>}^{W}C_{W}$$
  where ${\dot{W}}_{<9>}={\dot{E}}_{x,<9>}^{W}$.
10. HTX for coolant
  (24-1) $${\dot{H}}_{515}+{\dot{Q}}_{<10>}={\dot{H}}_{516}+{\dot{W}}_{x,<10>}$$
  (24-2) $$\left({\dot{E}}_{x,515}^{T}-{\dot{E}}_{x,516}^{T}\right)+\left({\dot{E}}_{x,515}^{P}-{\dot{E}}_{x,516}^{P}\right)+\left[T_{o}\left({\dot{S}}_{515}-{\dot{S}}_{516}\right)+{\dot{Q}}_{<10>}\right]={\dot{E}}_{x,<10>}^{W}$$
  (24-3) $$\left({\dot{E}}_{x,515}^{T}-{\dot{E}}_{x,516}^{T}\right)C_{10T}+\left({\dot{E}}_{x,515}^{P}-{\dot{E}}_{x,516}^{P}\right)C_{P}+\left[T_{o}\left({\dot{S}}_{515}-{\dot{S}}_{516}\right)+{\dot{Q}}_{<10>}\right]C_{S}+{\dot{Z}}_{<10>}=0$$
  where ${\dot{W}}_{x,<10>}={\dot{E}}_{x,<10>}^{W}$.
11. Coolant water tank
  (25-1) $${\dot{H}}_{518}+{\dot{Q}}_{<11>}={\dot{H}}_{512}$$
  (25-2) $$\left({\dot{E}}_{x,518}^{T}-{\dot{E}}_{x,512}^{T}\right)+\left({\dot{E}}_{x,518}^{P}-{\dot{E}}_{x,512}^{P}\right)+\left[T_{o}\left({\dot{S}}_{518}-{\dot{S}}_{512}\right)+{\dot{Q}}_{<11>}\right]=0$$
  (25-3) $$\left({\dot{E}}_{x,518}^{T}-{\dot{E}}_{x,512}^{T}\right)C_{11T}+\left({\dot{E}}_{x,518}^{P}-{\dot{E}}_{x,512}^{P}\right)C_{P}+\left[T_{o}\left({\dot{S}}_{518}-{\dot{S}}_{512}\right)+{\dot{Q}}_{<11>}\right]C_{S}+{\dot{Z}}_{<11>}=0$$
12. Pump 2
  (26-1) $${\dot{H}}_{512}+{\dot{Q}}_{<12>}={\dot{H}}_{513}+{\dot{W}}_{<12>}$$
  (26-2) $$\left({\dot{E}}_{x,512}^{T}-{\dot{E}}_{x,513}^{T}\right)+\left({\dot{E}}_{x,512}^{P}-{\dot{E}}_{x,513}^{P}\right)+\left[T_{o}\left({\dot{S}}_{512}-{\dot{S}}_{513}\right)+{\dot{Q}}_{<12>}\right]={\dot{E}}_{x,<12>}^{W}$$
  (26-3) $$\left({\dot{E}}_{x,512}^{T}-{\dot{E}}_{x,513}^{T}\right)C_{T}+\left({\dot{E}}_{x,512}^{P}-{\dot{E}}_{x,513}^{P}\right)C_{12P}+\left[T_{o}\left({\dot{S}}_{512}-{\dot{S}}_{513}\right)+{\dot{Q}}_{<12>}\right]C_{S}+{\dot{Z}}_{<12>}={\dot{E}}_{x,<12>}^{W}C_{W}$$
  where ${\dot{W}}_{<12>}={\dot{E}}_{x,<12>}^{W}$.

As explained in the previous section, the heat transfer rate obtained from the first law of thermodynamics can be used to obtain the irreversibility rate from the exergy balance equation. The chemical exergy of various gases [4,23] was only considered for the FCS. The hydrogen flow at the anode is considered a mass flow.

Twelve exergy cost-balance equations are obtained from 12 components, but there are 17 unknown unit costs in those equations, namely, C_1P_, C_2T_, C_3T_, C_4C_, C_5T_, C_6W_, C_7T_, C_8T_, C_9P_, C_10T_, C_11T_, C_12P_, C_T_, C_P_, C_C_, C_W_, and C_S_. To solve for these 17 unknown unit costs, we need the following auxiliary cost-balance equations for the junctions of various exergies:

Thermal exergy junction:

(27) $$\begin{array}{l} \left[\begin{array}{l} \left({\dot{E}}_{x,102}^{T}-{\dot{E}}_{x,103}^{T}\right)+\left({\dot{E}}_{x,104}^{T}-{\dot{E}}_{x,105}^{T}\right)+\left({\dot{E}}_{x,206}^{T}-{\dot{E}}_{x,207}^{T}\right)+\left({\dot{E}}_{x,509}^{T}-{\dot{E}}_{x,510}^{T}\right)+\\ \left({\dot{E}}_{x,510}^{T}-{\dot{E}}_{x,511}^{T}+{\dot{E}}_{x,513}^{T}-{\dot{E}}_{x,514}^{T}\right)+\left({\dot{E}}_{x,511}^{T}-{\dot{E}}_{x,508}^{T}\right)+\left({\dot{E}}_{x,515}^{T}-{\dot{E}}_{x,516}^{T}\right)+\left({\dot{E}}_{x,518}^{T}-{\dot{E}}_{x,512}^{T}\right) \end{array}\right]C_{T}\\ =\left[\left({\dot{E}}_{x,102}^{T}-{\dot{E}}_{x,103}^{T}\right)+\left({\dot{E}}_{x,104}^{T}-{\dot{E}}_{x,105}^{T}\right)\right]C_{2T}+\left({\dot{E}}_{x,206}^{T}-{\dot{E}}_{x,207}^{T}\right)C_{3T}+\left({\dot{E}}_{x,509}^{T}-{\dot{E}}_{x,510}^{T}\right)C_{5T}+\\ \left({\dot{E}}_{x,510}^{T}-{\dot{E}}_{x,511}^{T}+{\dot{E}}_{x,513}^{T}-{\dot{E}}_{x,514}^{T}\right)C_{7T}+\left({\dot{E}}_{x,511}^{T}-{\dot{E}}_{x,508}^{T}\right)C_{8T}+\left({\dot{E}}_{x,515}^{T}-{\dot{E}}_{x,516}^{T}\right)C_{10T}+\\ \left({\dot{E}}_{x,518}^{T}-{\dot{E}}_{x,512}^{T}\right)C_{11T} \end{array}$$

Mechanical exergy junction:

(28) $$\begin{array}{c} \left[\left({\dot{E}}_{x,101}^{P}-{\dot{E}}_{x,102}^{P}\right)+\left({\dot{E}}_{x,508}^{P}-{\dot{E}}_{x,509}^{P}\right)+\left({\dot{E}}_{x,512}^{P}-{\dot{E}}_{x,513}^{P}\right)\right]C_{P}=\left({\dot{E}}_{x,101}^{P}-{\dot{E}}_{x,102}^{P}\right)C_{1P}+\\ \left({\dot{E}}_{x,508}^{P}-{\dot{E}}_{x,509}^{P}\right)C_{9P}+\left({\dot{E}}_{x,512}^{P}-{\dot{E}}_{x,513}^{P}\right)C_{12P} \end{array}$$

Chemical exergy junction:

(29) $$C_{C}=C_{4C}$$

Work exergy junction:

(30) $$C_{W}=C_{6W}$$

Boundary:

(31) $$\begin{array}{l} -\left[\left({\dot{E}}_{x,101}^{T}-{\dot{E}}_{x,105}^{T}\right)+\left({\dot{E}}_{x,206}^{T}-{\dot{E}}_{x,207}^{T}\right)\right]C_{T}-\left[\left({\dot{E}}_{x,101}^{P}-{\dot{E}}_{x,105}^{P}\right)+\left({\dot{E}}_{x,206}^{P}-{\dot{E}}_{x,207}^{P}\right)\right]C_{P}-\left({\dot{E}}_{x,101}^{C}-{\dot{E}}_{x,105}^{C}\right)C_{C}\\ -T_{o}\left({\dot{S}}_{101}-{\dot{S}}_{105}+{\dot{S}}_{206}-{\dot{S}}_{207}\right)C_{S}=0 \end{array}$$

### 3.2. Energy Conservation and Exergy Balance of the FCS

Particular attention was paid to obtaining the energy conservation equation and exergy balance equation for the FCS, which consumes the chemical energy of hydrogen to produce electricity. The sum of the thermal exergy and the lost work in the exergy balance equation for the FCS is expressed as the chemical exergy (*P_therm_*), which is not converted to electric power. Since the chemical exergy of the fuel depends on the amount of condensation of the water product, the mole fraction of water in the water vapor produced during the electrochemical reaction of the FCS must be determined. By introducing the mole fraction (y) of liquid water in the total water vapor produced in the FCS, the chemical exergy of the fuel entering the FCS can be presented by Equation (32) [12].

(32) $$\begin{array}{ll} e_{x}^{CHE} & =(1-y)LHV+yHHV\\ & =LHV+yh_{fg} \end{array}$$

where *h_fg_* is the latent heat of H_2_O at a given temperature and *LHV* and *HHV* are the lower and higher heating values of water, respectively. In our study, the mole fraction of condensed water was taken as 0.395. Since the consumed chemical exergy in the FCS is presented by the difference between the input and output chemical exergy, the energy conservation equation of the FCS can be written as follows:

(33) $$P_{therm}={\dot{E}}_{x}^{CHE}(={\dot{n}}_{H_{2}}\cdot e_{x}^{CHE})-{\dot{E}}_{x,[7]}^{W}$$

The terms on the left-hand side (P_therm_) represent the chemical energy, which is not converted to electric power in the FCS. The mole flow rate of the fuel consumed in the FCS or the mole flow rate of hydrogen at the membrane level [24] can be obtained using the following equation.

(34) $${\dot{n}}_{H_{2}}=I\cdot N_{cell}/(2F)$$

where *I* is the current and *N_cell_* is the number of cells in the stack. The Faraday constant in Equation (34) is 26,801.48 A∙h/kmol.

The value of P_therm_ was substituted in the exergy balance equation using the following equation.

(35) $$P_{therm}={\dot{E}}_{x,<6>}^{D}+{\dot{E}}_{x}^{T}$$

The exergy destruction rate in the FCS <5> should be determined. The amount of heat transfer to the HTX for the FCS <6>, i.e., ${\dot{E}}_{x}^{T}$, was not considered to be the exergy destruction rate in the FCS.

### 3.3. Exergy Cost-Balance Equation for the Overall System

The overall exergy cost-balance equation of the system can be obtained by summing all the exergy cost-balance equations of the components and the cost-balance equations for the various exergy junctions and boundaries, which is presented by adding all the cost-balance equations of Equations (15-3)–(26-3) and (27)–(31). The exergy cost-balance equation for the overall system is as follows:

(36) $${\dot{E}}_{x}^{CHE}C_{o}+\left[\sum_{i}{\dot{Q}}_{<i>}\right]C_{S}+\sum_{i}{\dot{Z}}_{i}=\sum_{i}{\dot{E}}_{x,i}^{W}C_{W}$$

The second term on the left-hand side is the lost cost flow due to the heat transfer of all the components of the PEMFC system. However, the contribution of this term is negligible compared to other terms, such that Equation (36) can be simplified as follows:

(37) $${\dot{E}}_{x}^{CHE}C_{o}+\sum_{i}{\dot{Z}}_{<i>}=\sum_{i}{\dot{E}}_{x,<i>}^{W}C_{W}$$

In the case of a thermal energy system that produces one product, such as electricity, it is known that the unit cost of the product can be obtained using Equation (37) [16]. However, a detailed thermoeconomic analysis is needed to evaluate the costing process of the system and the loss of monetary flow in a particular process or device. The unit cost of a product estimated by some type of thermoeconomic method must be equal to the unit cost calculated using Equation (37).

## 4. Calculation Results and Discussion

The energy, exergy, and thermoeconomic analyses were conducted based on the experimental data obtained from a hydrogen-fueled 1-kW PEMFC system operated at the Korea Institute of Energy Research, Korea [12]. The PEMFC system consists of 24 cells in the stack, with an effective cell area of 220 cm^2^, producing 820 W of electricity under 100% current load. The measured thermodynamic properties, such as molar flow rate, temperature, and pressure, as well as the calculated enthalpy and entropy flow rates and total exergy flow rates at the various inlets and outlets of the components, are presented in Table 1 considering it was operated under a 100% current load. A dataset for the mole flow rate, temperature, and pressure was obtained by averaging 300 data points measured over 5 min at 1-s intervals. 

Table 2 lists the energy conservation for each component of the PEMFC system operating at full load conditions using the data shown in Table 1. The components of the FCS are not listed in this table because they have no material flow. The second and third columns show the enthalpy flows into and out of each component, respectively. The fourth and fifth columns show the workflow rate and thermal interaction of the components with their environment. The null values of the sum of the incoming and outgoing enthalpy flow, heat, and workflow rates indicate that energy conservation is maintained in each component. The plus sign in the heat transfer rate indicates the heat flow from the component to the environment or another component, thus increasing the entropy production of the component. This is not the traditional symbolic convention for heat transfer that can be obtained from the first law of thermodynamics given in Equation (1). The sixth column shows the irreversibility rate for each component calculated from the difference between the outgoing and incoming entropy flows. The seventh column represents the total irreversibility of the component, which is the sum of the irreversibility originating from the heat transfer and the irreversibility due to the entropy flow. The largest irreversibility occurs in the HTX for the coolant (10), which is approximately 310.6 kJ/h due to the heat transfer interaction between the component and the environment. 

The net flow rates of exergies crossing the boundary of each component in the PEMFC system under a 100% current load are listed in Table 3. A positive exergy value indicates the exergy flow rate of the ‘product’ (the right side of Equation (7)), while the negative values indicate the exergy flow rate of the ‘resources’ or ‘fuel’ (the left side of Equation (7)). Irreversibility acts as a “product” in the exergy balance equation, as can be clearly seen in Equation (7). The irreversibility rates of each component, shown in the sixth column, were obtained from the exergy balance equation in Equation (7). The exergy balance of the FCS in the seventh row indicates a stack-level efficiency of approximately 51.6%. The irreversibility rate of each component can be obtained from Equation (7) without any information about the heat transfer rate. The irreversibility value matched well with those calculated by the Gouy–Stodola theorem presented in Table 2. One exception is the cathode component, where the molar flow at the inlet is different from the molar flow at the outlet. The negative irreversibility rates of the cathode and the HTX in the FCS indicate that heat flows into the components, resulting in a decrease in the rate of entropy generation. As shown in Table 2 and Table 3, the highest irreversibility (310.6 kJ/h) occurs in the HTX for the coolant <10> due to heat transfer to the environment. The sum of the exergy flow rates of products and resources is zero for each component, indicating that the perfect exergy balance has been met. 

The irreversibility rate for the HTX for the FCS <5> is −258.09 kJ/h, which is significantly negative. Assuming that the HTX for the FCS is in thermal contact with the FCS stack, with a temperature of T_R_ = 333.2 K, the irreversibility rate is calculated as follows:

$$\begin{array}{c} {\dot{I}}_{\left[5\right]}=T_{o}\left({\dot{S}}_{510}-{\dot{S}}_{509}\right)-T_{o}{\dot{Q}}_{R}/T_{R}-{\dot{Q}}_{R}\left(1-T_{o}/T_{R}\right)=2484.490-2742.579\\ =-258.09\;kJ/h \end{array}$$

which is the same as the irreversibility in Table 3 (See Appendix A).

Negative values of the irreversibility rate also occurred in the generator and absorber of LiBr-water absorption refrigeration systems [6] and in the heat sink of the ground-source heat pump system [17]. Negative irreversibility occurs in components where the rate of heat input into the component is greater than the rate of irreversibility due to the entropy flowing out and the entropy flowing in. When a large amount of heat is introduced into a component, the entropy increases, causing the irreversibility of the component to be negative. 

Table 4 shows the unit cost of the primary product in each component, as shown in the exergy cost-balance equations. The unit cost of exergy was obtained by solving the 17 cost-balance equations simultaneously for the components and junctions. A hydrogen unit cost of $25/GJ ($3.0/kg H_2_) was used in this calculation. The input molar flow rate of hydrogen was taken as the consumed hydrogen in the FCS (0.00196 kmol/h) rather than the molar flow rate of hydrogen coming in at 206 state point (0.00275 kmol/h). The mole fraction of water condensate at the GDL layer was taken to be 0.35, which is a typical value for the normal operation of the PEMFC system [12]. The electrical unit cost of C_6W_ ($270.3/GJ) was obtained by adjusting the irreversibility value of the FCS to match the system production cost obtained using Equation (37). The unit cost of entropy destruction (C_S_) was −$4483.0/GJ.

In Table 5, the initial investment costs for all components are also listed in the seventh column of the table. The total investment cost of the PEMFC system is approximately $23,800, which is similar to the total investment cost of $23,424 for a 1-kW high-temperature PEMFC [25]. The sixth column of this table lists the investment cost flow rate for each component, which can be calculated using Equation (13). The cost flow rate for each component required to solve the cost-balance equation acts as an input cost (negative sign), as shown in the table. 

The cost flow rate for each exergy of each component at a 100% current load is given in Table 5. For example, the second row in Table 5 numerically expresses the exergy cost-balance equation for the air blower, as follows:

(15-3’) $$\begin{array}{l} {\dot{E}}_{x,[1]}^{W}C_{W}+\left({\dot{E}}_{x,102}^{T}-{\dot{E}}_{x,101}^{T}\right)C_{T}+\left({\dot{E}}_{x,102}^{P}-{\dot{E}}_{x,101}^{P}\right)C_{1P}+\left({\dot{E}}_{x,102}^{C}-{\dot{E}}_{x,101}^{C}\right)C_{C}\\ \;+\left[T_{o}\left({\dot{S}}_{102}-{\dot{S}}_{101}\right)-{\dot{Q}}_{\left[1\right]}\right]C_{S}-{\dot{Z}}_{\left[1\right]}=0 \end{array}$$

The cost flow rate can be calculated using the exergy flow rates shown in Table 3 and the unit costs of the primary product listed in Table 4. The same sign convention for the cost flow rates related to the products and resources was used in the case of exergy balances shown in Table 2. The monetary cost flow rate and the lost cost flow rate due to the entropy production rate in a component, which are negative values, are consumption costs, while the cost flow rate for the electricity produced in the FCS <5>, which is a positive value, is a production cost. The fact that the sum of the cost flow rates of each component becomes zero shows that all the cost-balance equations for the components are satisfied. 

As can be seen in Table 5, the cost flow rate to the net electricity production rate is about 0.79 $/h, and the net electricity production rate is about 2923.60 kJ/h, as shown in Table 3. The estimated electricity unit cost from these results is approximately $270.20/GJ. As shown in Table 5, the total lost cost flow rate is approximately 0.455 $/h, which is about 58% of the total cost flow rate of the initial investment, i.e., 0.778 $/h. In particular, capital losses incurred by the HTX for the coolant (10) amount to $1.392/h, indicating that this equipment requires a modification of the component. Heat transfer from the HTX to the environment by forced convection of air must be recovered by water flow to improve the performance of the PEMFC system. As shown in Table 5, the total investment for a 1-kW PEMFC system is $23,786.2, and this high initial investment cost results in a high unit cost of electricity produced by the system, which is $0.2703/MJ ($0.973/kWh). 

**Figure 2:**
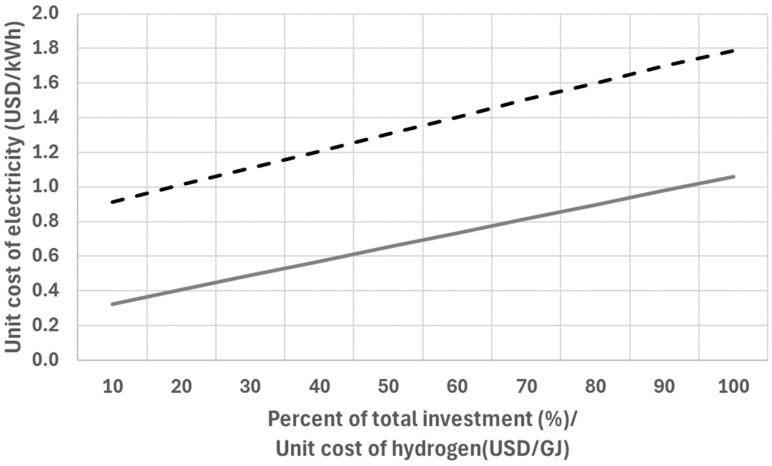
The unit cost of electricity depending on the investment cost of the system with a fixed hydrogen cost ($25/GJ: straight line) and the unit cost of electricity depending on the hydrogen cost with a fixed investment cost ($23,786: dotted line).

As shown in Figure 2, the unit cost of power produced by a PEMFC system drops linearly as the total investment cost of the PEMFC system decreases. For competitive electricity costs, the total investment cost of a 1-kW PEMFC system must be reduced to one-twentieth of the current total investment cost. The high cost of electricity produced by the PEMFC is due to the cost of hydrogen. The unit cost of hydrogen produced through methane gas reforming using a nuclear reactor is as low as $10.0/GJ [26]. However, the unit costs of hydrogen produced through water electrolysis using renewable energy sources such as wind and photovoltaic are estimated at $80.4/GJ [27] and $104.1/GJ [28], respectively. Figure 2 also shows how the unit cost of electricity produced by a PEMFC increases with the cost of hydrogen when the initial investment is fixed.

## 5. Conclusions

Energy, exergy, and thermoeconomic analyses were conducted for a 1-kW hydrogen-fueled proton-exchange membrane fuel system. A detailed energy, exergy, and thermoeconomic analysis of the PEMFC system shows that the HTX for the coolant has the greatest irreversibility and highest cost loss of all components. The irreversibility rate of the component was obtained using the Gouy–Stodola theorem, which states that the irreversibility rate depends on the difference in entropy flow rates entering and leaving the component and the heat transfer rate to the environment. Typically, the irreversibility rate of system components is obtained directly from the exergy balance equations without thermal interaction information. However, this method cannot determine the origin of entropy generation, which is a very important factor in system design and improvement. The irreversibility that occurred in the HTX for the coolant when using the Gouy–Stodola theorem is due to the forced convection of air. To increase the efficiency of a PEMFC system, another cooling system must be used to recover heat transfer to the environment. In residential applications of PEMFC systems [1,6], the heat gained in cooling the stack is recovered by the water stream.

## Figures and Tables

**Table 1:** Measured property values and calculated enthalpy, entropy, and exergy flow rates at various state points in the hydrogen-fueled 1-kW PEMFC under a 100% current load.

States	${\dot{n}}_{i}$ (kmol/h)	P (kPa)	T (K)	$\dot{H}$ (kJ/h)	$\dot{S}$ (kJ/h/K)	${\dot{E}}_{x}$ (kJ/h)
101	0.0846	100.90	299.40	3.0821	0.471	−1.186
102	0.0846	113.36	305.99	19.340	0.443	23.461
103	0.0846	105.40	322.14	59.280	0.621	10.214
104	0.0748	102.28	332.99	76.067	0.540	35.345
105	0.0748	102.42	312.52	31.335	0.407	30.376
206	0.0275	103.98	299.97	1.443	0.005	0.0
207	0.0079	102.05	326.10	6.368	0.020	0.002
508	4.8295	99.80	325.69	19,136.736	64.053	435.754
509	4.8295	143.63	325.59	19,103.614	63.940	436.496
510	4.8295	122.48	333.13	21,846.193	72.273	694.585
511	4.8295	110.48	326.14	19,301.256	64.555	450.631
512	2.2926	102.35	303.10	5185.052	17.998	7.057
513	2.2926	114.85	303.03	5173.442	17.958	7.378
514	2.2926	105.72	331.47	10,082.861	33.445	299.545
515	2.2926	103.72	331.47	10,082.791	33.445	299.463
516	2.2926	101.75	303.05	5176.401	17.970	6.891
518	2.2926	100.75	302.05	5003.788	17.400	4.340

**Table 2:** Energy conservation for each component in the PEMFC system.

	Enthalpy Inflow	Enthalpy Outflow	Workflow	Heat Flow	$T_{o}({\dot{S}}_{out}-{\dot{S}}_{in})$	Irreversibility Rate
Air blower	−3.082	19.340	−16.258	0.0	−8.389	−8.389
Anode	−1.443	6.368	0.0	−4.925	4.648	−0.277
Cathode	−59.280	76.067	0.0	−16.787	−24.097	−40.884
Humidifier	−95.408	90.615	0.0	4.793	13.484	18.277
HTX for the FCS	−19,103.614	21,486.193		−2742.579	2484.490	−258.089
HTXDI	−27,019.635	29,384.117	0.0	−2364.482	2316.269	−48.213
DIWT	−19,301.256	19,136.736		164.521	−149.644	14.877
Pump 1	−19,136.736	19,103.614	−5.503	38.625	−33.864	4.762
HTXWC	−10,082.791	5176.401	−18.000	4924.390	−4613.819	310.572
Hot WT	−5003.788	5185.052	0.0	−181.264	178.548	−2.716
Pump 2	−5185.052	5173.442	−0.645	12.255	−11.931	0.324

The unit is kJ/h. The entropy generation rate was calculated using the second law of thermodynamics, and the irreversibility rate was calculated using the Gouy–Stodola theorem.

**Table 3:** Exergy balance for each component in the hydrogen-fueled 1-kW PEMFC at a 100% current load. The irreversibility rate was calculated using the exergy balance equation given in Equation (7).

Component	Net Exergy Flow Rates (kJ/h)				Irreversibility
	${\dot{E}}_{x}^{W}$	${\dot{E}}_{x}^{CHE}$	${\dot{E}}_{x}^{T}$	${\dot{E}}_{x}^{P}$	Rate (kJ/h)
Air blower	−16.258	0.0	0.244	24.403	−8.389
Anode	0.0	0.0	1.622	−1.620	−0.002
Cathode	0.0	0.0	31.651	−6.521	−25.131
Humidifier	0.0	0.0	−1.381	−16.834	18.215
HTX in the FCS	0.0	0.0	259.935	−1.845	−258.090
FCS	2923.596	−5666.175	(2642.475)	0.0	100.104
HTX DI			49.638	−1.425	−48.213
DIWT	0.0	0.0	−13.945	−0.932	14.877
Pump 1	−5.503	0.0	−3.083	3.824	4.762
HTXWC	−18.0	0.0	−292.490	−0.082	310.572
Hot WT			2.650	0.066	−2.716
Pump 2	−0.645		−0.196	0.518	0.324
Total	2883.19	−5666.175	(2677.12)	−0.448	106.313

**Table 4:** Unit cost of various exergies in the cost-balance equations for the PEMFC system (the unit is USD/MJ).

**C_1P_**	**C_2T_**	**C_3T_**	**C_4C_**	**C_5T_**	**C_6W_**	**C_7T_**
−0.8955	−230.52	102.99	−86,238.0	−4.173	0.2703	−3.9587
**C_8T_**	**C_9P_**	**C_10T_**	**C_11T_**	**C_12P_**	**C_T_**	**C_P_**
5.80	94.494	−4.790	2.065	52.719	114.76	12.759
**C_C_**	**C_W_**	**C_S_**				
−86,238.0	0.2703	−4.483				

**Table 5:** Exergy cost-balance for each component in the hydrogen-fueled 1-kW PEMFC at a 100% current load. The unit is USD/h.

Component	Electricity Cost Flow	Thermal Exergy Cost Flow	Mechanical Exergy Cost Flow	Lost Cost Flow	Invest Cost Flow	Investment (USD)
Air blower	−0.004	0.028	−0.022	0.038	−0.039	1431.5
Anode		0.167	−0.021		−0.146	5332.4
Cathode	−3.515 *	3.632	−0.083	0.113	−0.146	5332.4
Humidifier		0.318	−0.215	−0.082	−0.022	800.0
HTX in the FCS		−1.085	−0.024	1.157	−0.049	1775.3
FCS	0.790			−0.449	−0.342	7856.6
HTX DI		−0.197	−0.018	0.216	−0.001	52.2
DIWT		0.081	−0.012	−0.067	−0.002	84.2
Pump 1	−0.001	−0.354	0.361	−0.001	−0.005	168.4
HTXWC	−0.005	1.401	−0.001	−1.392	−0.003	111.1
Pump 2		−0.022	0.027	−0.0	−0.005	168.4
Hot WT		0.005	0.001	0.012	−0.018	673.7
Total	−2.735 (0.78+)	3.974	−0.007	−0.455	−0.778	23,786.2

* Chemical exergy and + net electrical exergy.

## Data Availability

The original contributions presented in the study are included in the article, further inquiries can be directed to the corresponding author/s.

## Nomenclature

C	unit cost
${\dot{C}}_{i}$	annualized cost
$C_{i}$	initial investment cost
$C_{o}$	unit cost of fuel
$e_{x}$	exergy per unit mass
${\dot{E}}_{x}$	exergy flow rate
h	enthalpy per unit mass
$\dot{H}$	enthalpy flow rate
$h_{fg}$	latent heat of water
I	electric current
$\dot{I}$	irreversibility rate
$\dot{m}$	mass flow rate
$\dot{n}$	mole flow rate
$N_{cell}$	number of stacks
$\dot{Q}$	heat flow rate
s	entropy per unit mass
$\dot{S}$	entropy flow rate
${\dot{S}}_{gen}$	entropy generation rate
T	temperature
$T_{o}$	ambient temperature
$\dot{W}$	workflow rate
$\dot{Z}$	capital cost flow rate
**Greek letters**	
δ	annual operating hours
ϕ	maintenance cost factor
**Superscripts**	
C	chemical
CHE	fuel
D	exergy destruction
P	mechanical
T	thermal
W	work or electricity
**Subscripts**	
C	chemical
cv	control volume
o	reference point
P	mechanical
R	thermal reservoir
S	entropy
T	thermal
W	work or electricity

## Abbreviations

CCHP	combined cooling, heating, and power
CHP	cooling, heating, and power
CRF	capital recovery factor
DI	deionized
F	Faraday constant
FCS	fuel cell stack
HHV	higher heating value
LHV	lower heating value
PEMFC	proton-exchange membrane fuel cell
HTX	heat exchanger
PWF	present worth factor
SV	salvage value

## Appendix A

Consider a thermal system (or a component) that is in thermal contact with ambient temperature T_o_ and another heat reservoir of temperature *T_R_*. Assuming that the system has a heat source, the first law of thermodynamics is written as follows:

(A1) $$\frac{dE_{cv}}{dt}={\dot{Q}}_{source}+{\dot{Q}}_{o}+{\dot{Q}}_{R}+\sum_{in}{\dot{m}}_{i}h_{i}-\sum_{out}{\dot{m}}_{i}h_{i}-{\dot{W}}_{cv}$$

where ${\dot{Q}}_{o}$ and ${\dot{Q}}_{R}$ are the heat transfer interactions with the environment and thermal reservoir at temperatures of T_o_ and *T_R_*, respectively. In Equation (A1), ${\dot{W}}_{cv}$ is the workflow rate, $h_{i}$ is the enthalpy per mass, and ${\dot{m}}_{i}$ is the mass flow rate.

The second law of thermodynamics for the system is given by the following equation:

(A2) $$\frac{dS_{cv}}{dt}=\sum_{in}{\dot{m}}_{i}s_{i}-\sum_{out}{\dot{m}}_{i}s_{i}+\frac{{\dot{Q}}_{o}}{T_{o}}+\frac{{\dot{Q}}_{R}}{T_{R}}+{\dot{S}}_{gen}$$

where *S_i_* is the entropy per mass and ${\dot{S}}_{gen}$ is the entropy generation rate.

We can obtain the heat transfer interaction with the environment, i.e., ${\dot{Q}}_{o}$, from the second law of thermodynamics [18]. In such a case, Equation (A2) can be written as follows:

(A3) $${\dot{Q}}_{o}=T_{o}\left(\sum_{out}{\dot{m}}_{i}s_{i}-\sum_{in}{\dot{m}}_{i}s_{i}\right)+T_{o}\frac{dS_{cv}}{dt}-\frac{T_{o}}{T_{R}}{\dot{Q}}_{R}-T_{o}{\dot{S}}_{gen}$$

Substituting ${\dot{Q}}_{o}$ in Equation (A1), we obtain the following combined form of the first and second laws of thermodynamics.

(A4) $${\dot{Q}}_{source}+T_{o}\left(\sum_{out}{\dot{m}}_{i}s_{i}-\sum_{in}{\dot{m}}_{i}s_{i}\right)+\left(\sum_{in}{\dot{m}}_{i}h_{i}-\sum_{out}{\dot{m}}_{i}h_{i}\right)+{\dot{Q}}_{R}\left(1-\frac{T_{o}}{T_{R}}\right)-\frac{d}{dt}\left({\dot{E}}_{cv}-T_{o}{\dot{S}}_{cv}\right)-T_{o}{\dot{S}}_{gen}={\dot{W}}_{cv}$$

Equation (A4) can be rearranged using the definition of exergy given in Equation (6), as follows:

(A5) $${\dot{Q}}_{source}+\left(\sum_{in}{\dot{m}}_{i}e_{x,i}-\sum_{out}{\dot{m}}_{i}e_{x,i}\right)+{\dot{Q}}_{R}\left(1-\frac{T_{o}}{T_{R}}\right)-\frac{d}{dt}\left({\dot{E}}_{cv}-T_{o}{\dot{S}}_{cv}\right)-T_{o}{\dot{S}}_{gen}={\dot{W}}_{cv}$$

If we substitute the term $-T_{o}{\dot{S}}_{gen}$ from Equation (A3) into Equation (A4), we can see that Equation (A4) becomes the first law of thermodynamics.

The second law of thermodynamics provides the following lost work rate or irreversibility rate.

(A6) $$T_{o}{\dot{S}}_{gen}=\dot{I}=T_{o}\left(\sum_{out}{\dot{m}}_{i}s_{i}-\sum_{in}{\dot{m}}_{i}s_{i}-\frac{{\dot{Q}}_{o}}{T_{o}}-\frac{{\dot{Q}}_{R}}{T_{R}}\right)+T_{o}\frac{dS_{cv}}{dt}$$

For steady-state, the exergy-balance equation for any component can be obtained by substituting Equation (A6) to Equation (A5). That is:

(A7) $${\dot{Q}}_{source}+\left(\sum_{in}{\dot{m}}_{i}e_{x,i}-\sum_{out}{\dot{m}}_{i}e_{x,i}\right)+T_{o}\left[\sum_{in}{\dot{m}}_{i}s_{i}-\sum_{out}{\dot{m}}_{i}s_{i}+\left({\dot{Q}}_{o}+{\dot{Q}}_{R}\right)/T_{o}\right]={\dot{W}}_{cv}$$

Equation (A7) indicates that regardless of the temperature of the thermal reservoir, heat transfer between the thermal reservoir and the component is important for the irreversibility of the component.
